# Effect of Recombinant Helicobacter Outer Membrane Protein H (HopH) on Nitric Oxide Production by Peripheral Macrophage in BALB/c Mice

**Published:** 2019

**Authors:** Masoumeh Navidinia, Neda Soleimani, Narges Bodagh Abadi

**Affiliations:** 1. Faculty of Allied Medical Sciences, Shahid Beheshti University of Medical Sciences, Tehran, Iran; 2. Departments of Microbiology and Microbial Biotechnology and Biotechnology, Faculty of Life Sciences and Biotechnology, Shahid Beheshti University, Tehran, Iran; 3. Department of Genetics, Faculty of Biological Sciences, Tarbiat Modares University, Tehran, Iran

**Keywords:** *Helicobacter pylori*, Macrophages, Recombinant HopH

## Abstract

**Background:**

Some products of bacteria are reported as an immunomodulator. The *Helicobacter pylori* (*H. pylori*) outer membrane proteins play an important role in stimulation of immune system. The present study was performed to determine the *in vitro* effect of recombinant HopH of *H. pylori* on Nitric Oxide (NO) production and viability of mouse peritoneal macrophages.

**Methods:**

*H. pylori* recombinant HopH was produced in this study. Mice peritoneal macrophages were purified and cultured. Different concentrations of recombinant HopH were used for stimulation of macrophages in order to evaluate NO production. The cell viability was detected by MTT assay. NO amounts released in to the supernatants of cultured macrophages and LPS-stimulated macrophages (10 *μg/ml*) were detected by Griess reagent.

**Results:**

Results demonstrated that the suppressive effect of high concentrations of recombinant HopH on NO release and the stimulation effect of protein was shown in 15 *µg/ml*, compared to the control group. NO stimulation was significant in all the concentrations of LPS stimulated with HopH groups.

**Conclusion:**

According to our findings, recombinant HopH has a toxic effect in high concentration on cell. So it can be an anticancer candidate.

## Introduction

Macrophage belongs to the myeloid lineage and plays a key role in inflammation in host defense 1. This is an important component of natural immunity involved in elimination of microbial pathogens and inhibition of tumor growth. To carry out their functional activities, macrophages must become activated. It is known that these cells can be activated by lymphokines and bacterial products 2. *Helicobacter pylori (H. pylori) is* a specific pathogen of human stomach being colonized at least in half of the world population. The occurrence of this infection is strongly linked to the economic and social conditions 2–5. *H. pylori* is connected to the gastric epithelial cells *via* surface receptors, helicobacter outer membrane protein H (HopH) or outer inflammatory protein A which is one of the bacterial outer membrane proteins that is involved in inflammatory processes 6–8.

The HopH is a virulence factor with high antigenic properties that leads to the elevation of serum IL-8 9–12. This protein has previously been indicated to play a role in adhesion and colonization of *H. pylori* 13. Nitric Oxide (NO) which is produced by macrophages is one of the most important immune mediators against *H. pylori*. Macrophages defend against bacteria in the gastric mucus by different ways such as cytokines production as inducers of nitric oxide and active oxygen mediator production. For instance, *H. pylori* would be inhibited by induced macrophages. The stimulatory components of bacteria that play the defending role against immune responses and the parts that induce an effective response against bacteria in macrophages still need elucidation. The aim of the present study was to evaluate inflammatory effect of recombinant outer membrane protein of *H. pylori* (HopH) on viability and capability of nitric oxide production of mouse peritoneal macrophages.

## Materials and Methods

### Induction and expression of recombinant protein HopH

Pet28a vector was used for cloning and expression. The *hopH* gene was amplified by PCR (Polymerase Chain Reaction). PCR products and vector Pet28a were digested by two enzymes BamHI and XhoI (Fermentas Co., Lithuania). The sequence of primers was 5′AAT CCATGGTCCACGCTGAAAGGAATGGG-3′ and reveres primer was 5′AGGGGATCCCACTTTAACCG CTAATTCAACAC-3′. The PCR mixture was prepared in a final volume of 25 *μl*. The amplification mixture consisted of template DNA (2 *μl*), 0.1 *μM* of the respective primers, 2.5 *μl* of a 10-fold concentrate PCR buffer, 200 *μM* of deoxynucleotide triphosphates, 2.5 *μM* MgCl2, and 1.5 *U* of Taq DNA polymerase (Cinna Gene). A thermocycler (Mastercycler gradient; Eppendorf, Hamburg, Germany) was programmed with the following parameters: after an initial denaturation for 5 *min* at 95*°C*, 30 cycles of amplification were performed with denaturation at 95.8*°C* for 1 *min*, annealing at 62*°C* for 1 *min*, and DNA extension at 72*°C* for 1 *min*, followed by a final extension at 72*°C* for 10 *min*. Ligation procedure was performed by using the T4 DNA Ligase (Fermentas Co., Lithuania) enzyme and was transformed to *Escherichia coli* (*E. coli)* DH5α as the cloning host.

In order to express recombinant protein HopH, the expressional host *E. coli* BL21 was used. For screening expression capability of bacteria with recombinant plasmid, they were grown on solid LB containing the kanamycin (Mast co., UK) antibiotics. Then, individual colonies were sub-cultured in 3 *ml* of liquid LB (Luria Bertani) medium (Merck KGaA, Darmstadt, Germany) containing 50 *μg/ml* kanamycin and incubated for a night at 37*°C* with rotational movement (200 *rpm*). Recombinant plasmids were induced with 1 *mM* isopropyl-beta-D-thiogalacto pyranoside (IPTG) (Fermentas Co., Lithuania) until the turbidity of medium reached 0.6–1 at 600 *nm*. Since His-tag was embedded in the vector and in accordance to designed primers, Ni-NTA affinity chromatography (CO BioVision California) was used for protein purification. To prepare cell lysate, precipitant of cultured *E. coli* was suspended in PBS and was broken by 10 cycles of sonication of the suspension for 40 seconds at 4*°C* and 90% speed. The resulting suspension was centrifuged at 4*°C*, 12000 *rpm* for 20 *min* and the supernatant was collected and passed through filters with 0.45 *μ* pore size. After crossing the supernatant, the column was washed with different buffers until the absorbance of column output reached zero at 280 *nm*. Finally, buffer wash was passed through the filter and the outputs of the column were collected separately. The resulting protein component was visualized on SDS-PAGE to verify its purity. In order to remove imidazole from the protein, exchange buffer dialysis was used in the presence of Phosphate-Buffered Saline (PBS) buffer (Merck Co., UK). Final concentration was determined by Bradford method.

### Isolating, culturing and stimulating macrophages

Female BALB/c mice of 6–8 weeks were purchased from the Pasteur Institute of Iran. For isolating peritoneal macrophages, 10 *ml* of cold culture medium RPMI-1640 (Sigma) was injected into peritoneal mice and collected afterward 14,15.

Macrophages isolated from 5 mice were pooled. After washing the cells, suspensions of 3×10^5^ *cells/ml* in RPMI medium (containing supplements as 2 *gr/L* of sodium bicarbonate, 2 *mM* L-glutamine, 100 *units/ml* penicillin, 100 *μg/ml* streptomycin and 10% fetal bovine serum) were seeded in each well of 96-well plates (Nunk) and incubated for 4 *hr* at 37*°C* in 5% CO_2_. During incubation, adherent cells (95% macrophages) clung to the bottom of plate and the non-adherent cells were removed by washing with 37*°C* PBS buffer. Concentration series of 15, 30, 60 and 125 *μg/μl* of recombinant protein HopH were used to stimulate the macrophages. In addition to the same culture and stimulation method in the second set, 10 *μg/ml* of Lipopolysaccharide (LPS) (obtained from Dr. Esmaili, Baghiyatalah University) were added to all the cultured macrophages as a general stimulant. Each experiment was performed in triplicates and un-stimulated macrophages were used as controls.

### MTT assay to assess the viability of macrophages

MTT [3-(4, 5-Dimethylthiazol-2-yl)-2, 5-Diphenyltetrazolium Bromide] assay was used for evaluation of macrophages viability in which reduction reflects the metabolic activity of the cells 12. For this purpose, after 48 *hr* of macrophage culture, 20 *μl* of MTT (5 *mg/ml* PBS) (Merck) was added to the wells and incubated for 4 *hr* at 37*°C*. After incubation, the supernatant was gently removed and 200 *μl* of DMSO (Sigma) was added to the wells in order to dissolve formazan crystals, and produce color. Plates were read at 570 *nm* absorption. The results were obtained by stimulation index (Stimulation Index: SI). SI calculated the test absorption to control absorption at 570 *nm* 12,16,17.

### Determining viability and survival of treated cells

Viability and survival of treated cells was determined using trypan blue staining. Separate cell groups treated with the recombinant protein were trypsinised and were washed, and afterward, the cells were stained with trypan blue and the amount of viable cells was determined by haemocytometer.

### Measurement of nitrite concentration

Nitrate amount from cultured macrophages was measured according to the Nathan and Stover method by using Griess reagent 10 After 48 *hr* of culturing, the supernatant of macrophages was collected and mixed with the Griess reagent in 96-well plates. After 15 *min*, the absorbance was measured at 450 *nm* using the micro plate absorbance reader (Multi Scan). Standard curve (1–200 *μm*) solution of sodium nitrite (NaNO_2_) was used to calculate the nitrite concentration 11.

### Statistical analysis

This study was an interventional study. Data on various recombinant proteins concentrations are presented as mean±SD by using Analysis of Variance (ANOVA) followed by Tukey test. Results were evaluated with SPSS ver 21 and Prism6 and p≤0.001 is interpreted as significant.

## Results

### Expression and purification of recombinant proteins HopH in the host E. coli BL21

**Figure 1.**
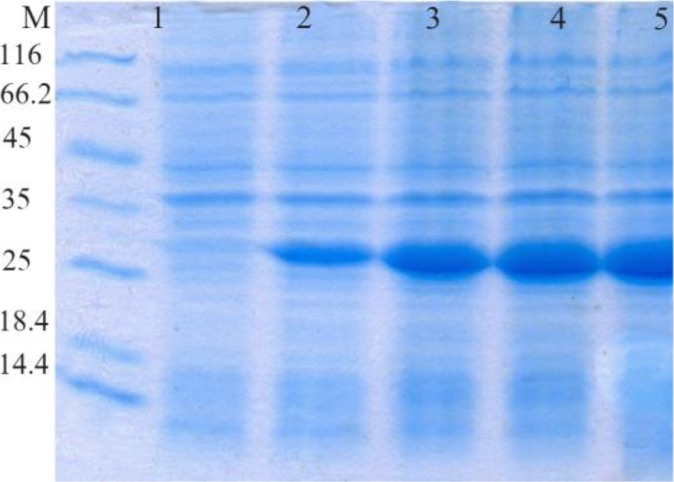
Evaluation of HopH protein expression on SDS-PAGE gel (12%): well M: marker with low weight, well 1: uninduced HopH, well 2: induced HopH by 0.1 *mM* IPTG within the first *hr*, well 3: induced HopH by 0.1 *mM* IPTG within the second *hr*, well 4: induced HopH by 0.1 *mM* IPTG within the third *hr*, well 5: induced HopH by 0.1 *mM* ITG within the fourth *hr*.

**Figure 2.**
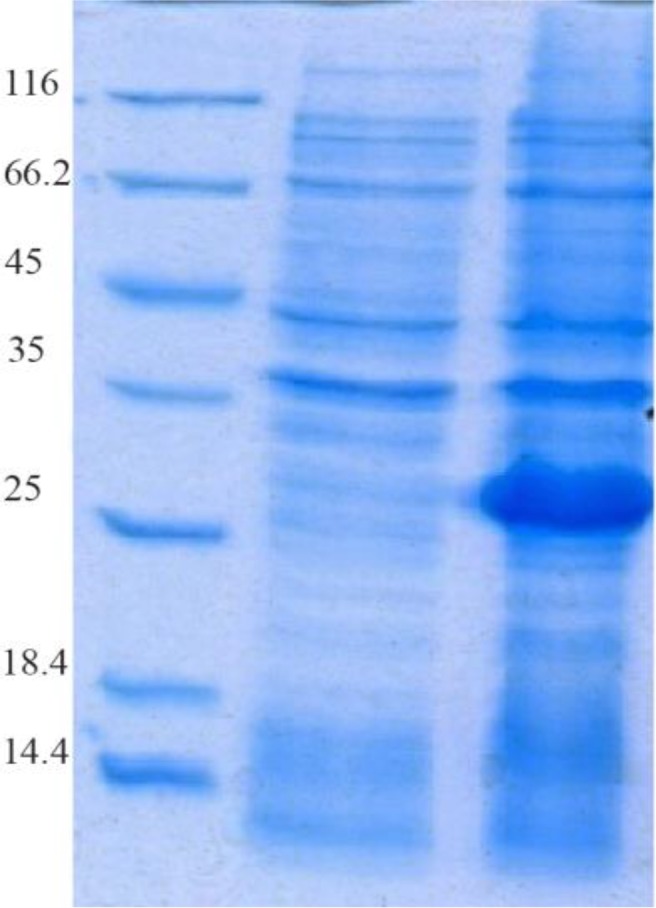
Evaluation of HopH protein expression on SDS-PAGE gel (12%): well M: marker with low weight, well 1: uninduced HopH, well 2: induced HopH by 0.1 *mM* IPTG within the fourth *hr*.

The SDS-PAGE represented the expression of a 34 *kDa* protein. According to measurements by Bradford method (with standard BSA) and spectrophotometry, extraction of 270 *μg/ml* protein deposits was obtained from one liter of bacteria culture (Figures 1 and 2).

### Proliferation assay

**Figure 3.**
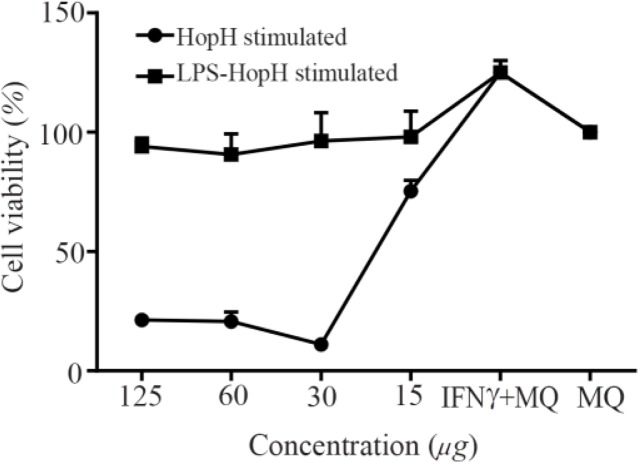
The viability of macrophages treated with different concentrations of HopH. Experiments were performed in triplicates; means± SDs are shown.

Macrophages were stimulated by LPS as a general inducer. Macrophages proliferation was evaluated by MTT assay after 24 *hr* of incubation and HopH treatment had significantly augmented the macrophages viability as compared with their negative control (Only macrophages culture) and LPS was used with HopH in treatment (Figure 3).

### Measurement of nitric oxide production

**Figure 4.**
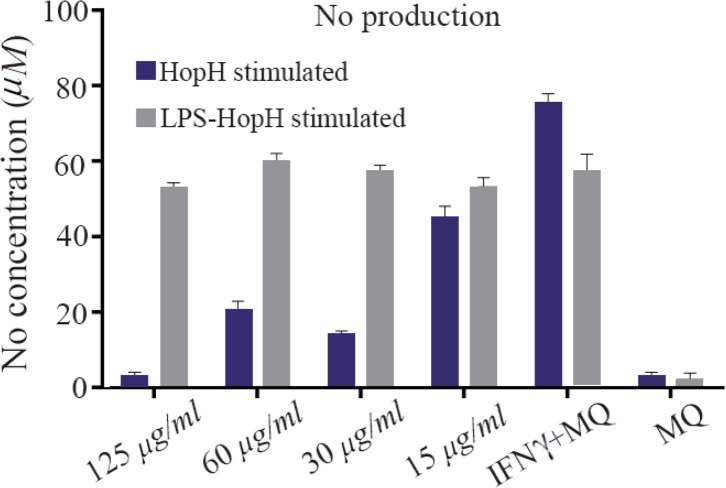
Nitric oxide production in macrophages exposed to different concentrations of HopH protein.

Levels of nitric oxide production in macrophages exposed to different concentrations of HopH are shown in figure 4. The macrophages were stimulated by LPS as a general inducer. The results show that NO production in the group exposed to 60 *μg/ml* without LPS was significantly higher than the group exposed to HopH with LPS (p<0.001). Statistical analysis showed that in both series, stimulated macrophages with 15 *μg/ml* concentrations of HopH are higher than other groups and the control group (p<0.001 for both). The result shows that HopH protein has a dose dependent manner and in high concentrations has toxic effect for cell but in low concentrations could stimulate the immune system.

## Discussion

Taking advantages of immunostimulator in order to increase host defense response is an alternative method for traditional therapy of various diseases by antibiotics. Numerous immunologic properties of microorganisms were studied by extraction and characterization of their antigens for vaccine production, diagnosis and epidemiologic studies. Recently, by expansion of knowledge in the field of host defense, infecting agents are destructed and dead by the NO and Reactive Oxygen Species (ROS) produced by macrophages. With regard to various functions of macrophages in pathogenesis and infection diseases, every modification in their functions could be useful as potential therapeutic strategies 18. Up to now, immune defense has been stimulated by different methods including bacterial by products or recombinant proteins. Yousefi *et al* studied the immunomodulatory effects of parsley oil. Their study results showed that nitric oxide production and macrophages activity were suppressed by this plant-derived substance 11. Shark cartilage effect on the levels of nitric oxide production and survival of murine peritoneal macrophages was studied by Haji moradi *et al* 13. Their results indicated that shark cartilage can increase the production of nitrite 13. Our previous study results on recombinant Bacterioferritin of *H. pylori* showed that this protein can stimulate immune system and produce nitric oxide 19. In this study, some research was done on microbial metabolite but no research on the effect of HopH recombinant protein of helicobacter was shown on activation of macrophage.

Using bioinformatics method, *hopH* gene cloning was optimized in *E. coli* by Teymournejad *et al* 18. Their results verify that cloned gene with engineered primers was correct. In this project, inflammatory recombinant outer membrane protein of *H. pylori* was synthesized.

In the present study, macrophages were treated by HopH protein in two series of induced and uninduced with LPS. Macrophages were stimulated by LPS as a general inducer. Results showed that macrophages treated with low concentrations of HopH had significantly higher viability and growth rate than the control group. NO production levels were significantly higher in series with the same concentration of HopH. This increase could be due to accession of macrophages growth, active cells or effect of HopH on the activity of macrophages. MTT results showed that viability and growth rates show differences in various groups. Therefore, 15 *μg/ml* of HopH caused induction in macrophages activity in NO production that in comparison with the same active cells and accession in NO production is due to augmentation of macrophages activity by HopH. This has a dose dependent manner. High dose of protein could be toxic for macrophage and low dose could stimulate immune system. In high dose, recombinant Bacterioferritin of *H. pylori* could be stimulated by immune system but HopH is toxic for cell 19.

## Conclusion

In conclusion, results of the present study indicate that recombinant HopH *H. pylori* have proper response in anticancer agent. These products are suggested as anticancer candidates and *in vivo* experiments could help us to reach this goal. Future studies for identification of signaling pathways and clarifying the role of these recombinant proteins are necessary.
